# 

**Published:** 1895-09

**Authors:** 


TABLE OF CONTENTS ON LAST ADVERTISING PAGE.

VOL. XI. SEPTEMBER, 1895. No. 3.

### TEXAS Medical Journal.

SUBSCRIPTION, $2.00 A YEAR, IN ADVANCE.
SINGLE COPIES, 25 CENTS.

PUBLISHED AT AUSTIN, TEXAS, BY

F. E. DANIEL, M. D., & S. E. HUDSON, M. D.,

EDITORS AND PROPRIETORS.

Entered at the Postoffice at Austin, Texas, as Second-class Mail Matter.

